# Extracellular vesicles from human breast cancer-resistant cells promote acquired drug resistance and pro-inflammatory macrophage response

**DOI:** 10.3389/fimmu.2024.1468229

**Published:** 2024-10-15

**Authors:** Patrick Santos, Caroline P. Rezende, Renan Piraine, Bianca Oliveira, Francielle B. Ferreira, Vinicius S. Carvalho, Rodrigo T. Calado, Matteo Pellegrini, Fausto Almeida

**Affiliations:** ^1^ Department of Biochemistry and Immunology, Ribeirão Preto Medical School, University of São Paulo, Ribeirão Preto, SP, Brazil; ^2^ Department of Medical Imaging, Hematology, and Oncology, Ribeirão Preto Medical School, University of São Paulo, Ribeirão Preto, Brazil; ^3^ Department of Molecular, Cell, and Developmental Biology, University of California, Los Angeles, CA, United States

**Keywords:** chemoresistance, tamoxifen, doxorubicin, immunomodulation, membrane transporters

## Abstract

**Introduction:**

Breast cancer is a significant public health problem around the world, ranking first in deaths due to cancer in females. The therapy to fight breast cancer involves different methods, including conventional chemotherapy. However, the acquired resistance that tumors develop during the treatment is still a central cause of cancer-associated deaths. One mechanism that induces drug resistance is cell communication via extracellular vesicles (EVs), which can carry efflux transporters and miRNA that increase sensitive cells’ survivability to chemotherapy.

**Methods:**

Our study investigates the transcription changes modulated by EVs from tamoxifen- and doxorubicin-resistant breast cancer cells in sensitive cells and how these changes may induce acquired drug resistance, inhibit apoptosis, and increase survivability in the sensitive cells. Additionally, we exposed human macrophages to resistant EVs to understand the influence of EVs on immune responses.

**Results:**

Our results suggest that the acquired drug resistance is associated with the ability of resistant EVs to upregulate several transporter classes, which are directly related to the increase of cell viability and survival of sensitive cells exposed to EVs before a low-dose drug treatment. In addition, we show evidence that resistant EVs may downregulate immune system factors to evade detection and block cell death by apoptosis in sensitive breast cancer cells. Our data also reveals that human macrophages in contact with resistant EVs trigger a pro-inflammatory cytokine secretion profile, an effect that may be helpful for future immunotherapy studies.

**Discussion:**

These findings are the first transcriptome-wide analysis of cells exposed to resistant EVs, supporting that resistant EVs are associated with the acquired drug resistance process during chemotherapy by modulating different aspects of sensitive cancer cells that coffer the chemoresistance.

## 1 Introduction

Human breast cancer is the most commonly diagnosed cancer worldwide and is a primary health problem to be addressed globally for future generations (1). Breast cancer is the major cause of cancer-related deaths owing to cancer among the female population, accounting for 11.7% of all new cases and 6.9% of new deaths in 2020, according to the Global Cancer Statistics (2). The survival rate of patients with breast cancer differs across regions worldwide. Survivability is constantly increasing in developed countries owing to investments in early detection technologies and access to modern medicines (3). Currently, breast cancer therapy involves different approaches, such as tumor removal surgery, radiotherapy, and conventional drugs, including traditional chemotherapeutics in combination with hormonal or immunotherapeutic agents (4).

Nonetheless, the toxicity of many drugs toward normal tissues and resistance to chemotherapy frequently used in breast cancer treatment are critical health concerns (5). The initial innate inability to respond to drug therapy, followed by acquired drug resistance, is the central cause of cancer-associated deaths (6). Acquired drug resistance involves many factors that cancer cells can manage to reduce drug efficacy, such as the abnormal activity of membrane transporters, a decrease in cell death processes, altered cellular metabolic signaling, deregulated protein expression, and interaction with receptors (7). More recently, another mechanism by which tumor cells avoid chemotherapeutic effects was orchestrated by extracellular vesicles (EVs) (8).

What was first thought to be cell garbage became a significant mechanism of communication between cells. Almost all cell types release EVs and mediate several cellular processes under homeostasis; however, EVs play an essential role in cancer as they serve as messengers in the tumor microenvironment to send different signals, such as signals to disrupt the immune system, during the promotion and progression of cancer (9). Fundamentally, EVs are lipid-bilayered vesicles with an endosomal or cellular origin that can carry a variety of molecules, including nucleic acids, lipids, carbohydrates, and proteins, via the bloodstream and other body fluids (10). Additionally, EVs can act as nano-carriers to enhance the anticancer effect of chemotherapeutic drugs (11). In the context of drug resistance, EVs can contain the drug itself as cargo, functioning as a drug delivery method to the extracellular space. Resistant cancer cell-derived EVs can carry drug transporters, such as P-glycoprotein, to promote drug efflux in nearby sensitive cells or even carry miRNAs to induce the expression of genes related to membrane transporters (12).

In this study, we combine the evaluation of crucial cellular processes, such as viability, proliferation, and cell death, with genome-wide transcriptomic analysis of breast cancer cell lines to investigate the influence of EVs released by resistant cancer cells on acquired drug resistance mechanisms in sensitive cells, which is a topic that remains unclear in the literature. We used two different breast cancer cell lines, MCF-7 as an epithelial and primary tumor model and MDA-MB-231 as a mesenchymal/high-mobility triple-negative model, to cover different aspects of cancer progression. We managed two human macrophage cultures to identify the ability of resistant EVs to induce phenotypic polarization in these cells after exposure. Our results indicate that EVs from resistant cells may transfer the acquired drug resistance phenotype to sensitive cells by upregulating several types of membrane transporters involved in drug resistance, resulting in increased cell viability and clonogenic survival in sensitive breast cancer cells exposed to EVs released by resistant cells. Resistant cancer cell-derived EVs may modulate the immune system by downregulating different immune factors. We observed that resistant cell-derived EVs polarized human macrophages to a pro-inflammatory phenotype, which represents a potential tool for immunotherapy.

## 2 Materials and methods

### 2.1 Cancer cells, macrophages and PBMC culture

The human triple-negative breast cancer cell line MDA-MB-231 was acquired from the American Type Culture Collection (ATCC, HTB-26) and cultured in RPMI-1640 medium supplemented with 1000 IU penicillin-streptomycin and 10% fetal bovine serum (FBS) in a humidified atmosphere at 37°C with 5% CO2. The human mammary gland-derived breast cancer cell line MCF-7 was acquired from Rio de Janeiro’s Cell Culture Bank and cultured in RPMI-1640 medium supplemented with 1000 IU penicillin-streptomycin and 10% FBS in a humidified atmosphere at 37°C with 5% CO_2_. The human monocyte cell line THP-1 was acquired from ATCC (TIB-202) and cultured in RPMI-1640 medium supplemented with 1000 IU penicillin-streptomycin and 10% FBS in a humidified atmosphere at 37°C with 5% CO_2_. Whole blood samples (10 mL/donor) were isolated from healthy female and male donors, without a defined age range, for experimental purposes only, following the principles proposed by the National Research Ethics Commission and Research Ethics Committee under the protocol CAAE 71085023.1.0000.5440. Human peripheral blood mononuclear cells (PBMCs) were isolated from whole blood samples by density gradient centrifugation using Histopaque^®^-1077 (MERCK, USA). The buffy coat containing PBMCs was transferred to a different tube to isolate CD14+/CD16- monocytes by negative selection using magnetic beads from a commercial classical monocyte isolation kit (Myltenyi Biotec, USA). The purification steps and macrophage differentiation are described in **Supplementary Table S1**. Purified CD14+/CD16- monocytes were plated in cell culture dishes containing RPMI-1640 medium supplemented with FBS and 10000 IU of penicillin-streptomycin. To induce the differentiation of monocytes into macrophages, 10 ng/mL of granulocyte-macrophage colony-stimulating factor (Peprotech, USA) was added to the monocyte culture for five days in a 37°C and 5% CO_2_ incubator. Tamoxifen (TAM) was acquired from AdipoGen Life Sciences (USA) and doxorubicin (DOX) was acquired from Sigma-Aldrich (USA).

### 2.2 Acquisition of the IC50 and viability of sensitive and resistant cells

The method chosen to evaluate the half-maximal inhibitory concentration (IC50) of sensitive and resistant cells was the reduction of resazurin, following the protocol described by Kumar (13). Briefly, sensitive or resistant cells of MCF-7 and MDA-MB-231 were seeded in 96-well plates, incubated for 24 h, and treated with a wide range of concentrations of TAM (0.62 to 160 μM) and DOX (0.08 to 20 μM) for 72 h. After the treatment, cells were incubated with a resazurin solution (88 μM) for 4 h, and the plates were read with 570 and 600 nm filters. Cell viability was calculated using the absorbance reads on the indicated equation according to the resazurin manufacturer’s protocol, in which cell viability was expressed as a percentage of live cells in each treatment condition compared with the control. Cell viability was observed by standard staining with fluorescein diacetate (FDA) and propidium iodide (PI) to differentiate viable from dead cells. Staining solutions were prepared to a final concentration of 80 μg/mL of FDA and 200 μg/mL of PI. Cells in contact with the staining solutions were incubated for 30 min at 37°C and then washed to remove the staining excess. After washing, the cells were observed under a fluorescence microscope using the filters according to the manufacturer’s protocol. The captured images were overlaid by NIS-Elements BR to create merged images.

### 2.3 Generation of drug-resistant breast cancer cell lines

TAM- and DOX-resistant breast cancer cell lines were developed following practical guidelines published by McDermott et al. (14). Using the previously calculated IC50 of each drug in MCF-7 and MDA-MB-231 sensitive cells, a high-level laboratory model of stable drug-resistant cell lines was generated by continuous exposure of sensitive cells to stepwise dose progression, following a comparative selection method based on flask cell confluence and cell viability via trypan blue staining. After obtaining the IC50 of TAM- and DOX-resistant cell lines, the fold resistance was determined as the ratio between the IC50 of the resistant cell line and the IC50 of the sensitive cell line. Cell lines with a fold resistance equal to or higher than 2 were considered sufficiently resistant to proceed with the other experiments.

### 2.4 Isolation, purification, characterization, and internalization of EVs

The differential ultracentrifugation method was used to isolate EVs, following the guidelines of Momen-Heravi (15). Briefly, the supernatants from the cell flasks or plate wells were centrifuged for 10 min at 1500 × g to remove dead cells, transferred to new tubes, and centrifuged for 30 min at 15,000 × g to remove cell debris and large particles. Finally, the supernatants were transferred to appropriate ultracentrifuge tubes and centrifuged at a high rotation speed of 100,000 × g for 70 min to isolate EVs. All centrifugations were performed at 4°C. The pellet of EVs was suspended in 100 μL of RNAse and DNAse-free ultra-pure water, following the guidelines suggested by the International Society of Extracellular Vesicles (ISEV) (16). Aliquots were prepared for characterization using transmission electron microscopy (TEM) and nanoparticle tracking analysis (NTA). The remaining suspended EVs were stored at -80°C. To observe the internalization of EVs upon contact with cancer cells, a lipophilic membrane stain was used to label the EVs following the manufacturer’s instructions. Previously isolated EVs were suspended in 200 μL of phosphate-buffered saline (PBS) with 1 μM of Vybrant Dil dye (Ex, 530 nm; Em, 580 nm, Thermo Fisher Scientific, USA) for 30 min at 37°C to obtain the labeling. The labeled EVs were centrifuged at 100,000 × g for 70 min and washed using another ultracentrifugation step in PBS. Labeled EVs were incubated with sensitive breast cancer cells for 24 h at 37°C and 5% CO2. Images were then captured in a bright field and under the appropriate fluorescence measurements in a Nikon Eclipse Ts2 with an attached Digital Sight 10 camera, followed by an overlay of the bright field and fluorescence images using ImageJ software to obtain merged images (17).

### 2.5 RNA extraction, library preparation, and sequencing

Messenger RNA (mRNA) was extracted from MCF-7 and MDA-MB-231 cell lysates using a spin column supplied with the RNeasy kit (QIAGEN, Germany). Both cell lines were co-cultured with TAM- and DOX-resistant EVs for 24 h. All extractions were performed in triplicate for each cell line following the manufacturer’s protocol by a single researcher to obtain high-quality mRNA. The mRNA capture and construction of 150 base pair double-stranded mRNA-Seq libraries were performed using Zymo-Seq RiboFree Total RNA (Zymo Research, USA). The protocol included the capture, elution, and fragmentation of mRNA, followed by synthesis of the first and second 150 strands. Illumina adapters were ligated to the ends of the mRNA-derived cDNA fragments, followed by library PCR amplification. The libraries were sequenced in the SP lane of an Illumina NovaSeq6000 sequencer.

### 2.6 Differential expression analysis of RNA-Seq

A quality control test was performed before alignment using FastQC software (18). All low-quality reads and Illumina adapters were removed by the 0.39 version of the Trimmomatic tool (19). Reads that passed the quality filter were aligned to the reference genome index (Homo sapiens Ensembl GRCh38, release 109) using STAR 2.7.0a alignment software (20) at the University of California, Los Angeles UNIX-based cluster. The alignment rate and total number of mapped reads are shown in **Supplementary Table S1**. Differential expression analysis was performed using the RStudio platform with 4.3.1 R (21). The DESeq2 package was used to detect differentially expressed genes (DEGs) by using negative binomial generalized linear models (22). The comparison between sensitive EVs vs. resistant EVs treatment for each cell line was estimated by the logarithmic fold-change log_2_ (sensitive vs. resistant). The lists with the DEGs of each condition were filtered using a 5% false discovery rate (FDR) cutoff and an absolute fold change > 1.5 for upregulated and downregulated genes.

### 2.7 Differential expressed genes’ function enrichment

The initial gene ontology (GO) slim analysis was performed using the DEGs lists of each condition in the web-based tool WebGestAlt (23) with a 1% FDR and Benjamini-Hochberg multiple test adjustment. To investigate the signaling pathways associated with the DEGs, we performed an over-representation analysis in GSEA software 4.3.2, with a 1% FDR cutoff, using the Molecular Signatures Database (24, 25). The online tool g:Profiler was used to understand the relationship between the major enriched signaling pathways reported in the prior analysis (26). Functional interactions between potential protein expression among DEGs were performed using STRING networks with a protein-protein interaction FDR of 5% (27).

### 2.8 Quantification of apoptosis after sensitive cells were exposed to resistant EVs

The detection of apoptotic cells was based on annexin V binding and PI uptake by flow cytometry using the Alexa Fluor^®^ 488 Annexin V/Dead Cell Apoptosis (Thermo Fisher Scientific, USA), following the manufacturer’s instructions. Briefly, MCF-7 and MDA-MB-231-sensitive cells were exposed to sensitive EVs as controls and to TAM- or DOX-resistant cells for 24 h. After exposure to EVs, the supernatant was removed from each well, and fresh media containing different concentrations of TAM or DOX, up to the previously calculated sensitive cell IC50, was incubated for another 72 h. The cells were harvested, stained with Annexin V-FITC and PI, and incubated for 15 min at room temperature. Annexin V-FITC/PI signals were assessed using a FACSCanto flow cytometer and analyzed using the FACSDiva software (BD Biosciences, USA).

### 2.9 Drug resistance-related genes expression assessment by qPCR

Total RNA was isolated from sensitive cells after 24 h of exposure to TAM- or DOX-resistant EVs using a spin column supplied in the RNeasy kit (QIAGEN, Germany), and cDNA was generated from 1 μg of RNA using the High-Capacity cDNA Reverse Transcription Kit (Applied Biosystems, USA), according to the manufacturer’s protocol. The conditions used in the qPCR reactions were performed using the Power SYBR™ Green PCR Master Mix (Applied Biosystems, USA) in an ABI 7500 FAST system (Applied Biosystems, USA) and following the manufacturer’s instructions. All samples were analyzed in triplicates. Relative expression of target genes was calculated by comparing threshold cycle (Ct) values of GAPDH as the housekeeping gene, according to the 2-ΔΔCT method (28). Acquired resistance (*ABCB1*, *ABCG2*, *TGFBR1*, *TGFBR2*, *TGFBR3*) and apoptosis-related genes (*BCL2*, *CD44*, *PIK3CA*, *PTEN*) were selected as target genes. The primers used are described in **Supplementary Table S2**.

### 2.10 Evaluation of clonogenic survival of breast cancer cells exposed to resistant EVs

The clonogenic survival of sensitive cells previously exposed to TAM- or DOX-resistant EVs was evaluated using a colony-forming assay using an *in vitro* cell protocol published by Franken (29). Briefly, MCF-7 and MDA-MB-231 cells were cultured with sensitive (control) or resistant EVs for 24 h. Each condition involved treatment with different concentrations of TAM or DOX for 72 h. The cells were resuspended, counted, and reseeded in a low-density manner (250 cells/well) in six-well plates for 7 days. The colonies were fixed with 6% (v/v) glutaraldehyde and stained with 0.5% (w/v) crystal violet for 30 min. Colonies containing 50 cells were counted.

### 2.11 Cytokine production of macrophages after exposure to resistant derived EVs

Interleukin (IL)-1β, IL-10, interferon-gamma (IFN-γ), and tumor necrosis factor (TNF)-α levels from macrophages supernatant after exposure of sensitive or resistant derived EVs for 24 h were measured using a commercial enzyme-linked immunosorbent assay (ELISA) kit following the manufacturer’s instructions (BD Biosciences, USA). The results were measured at an optical density of 450 nm using a SpectraMax 190 spectrophotometer (Molecular Devices). The results are reported as pg/mL of experimental and biological triplicates for each condition.

## 3 Results

### 3.1 Generation of drug resistant breast cancer cell lines

**Figure 1:**
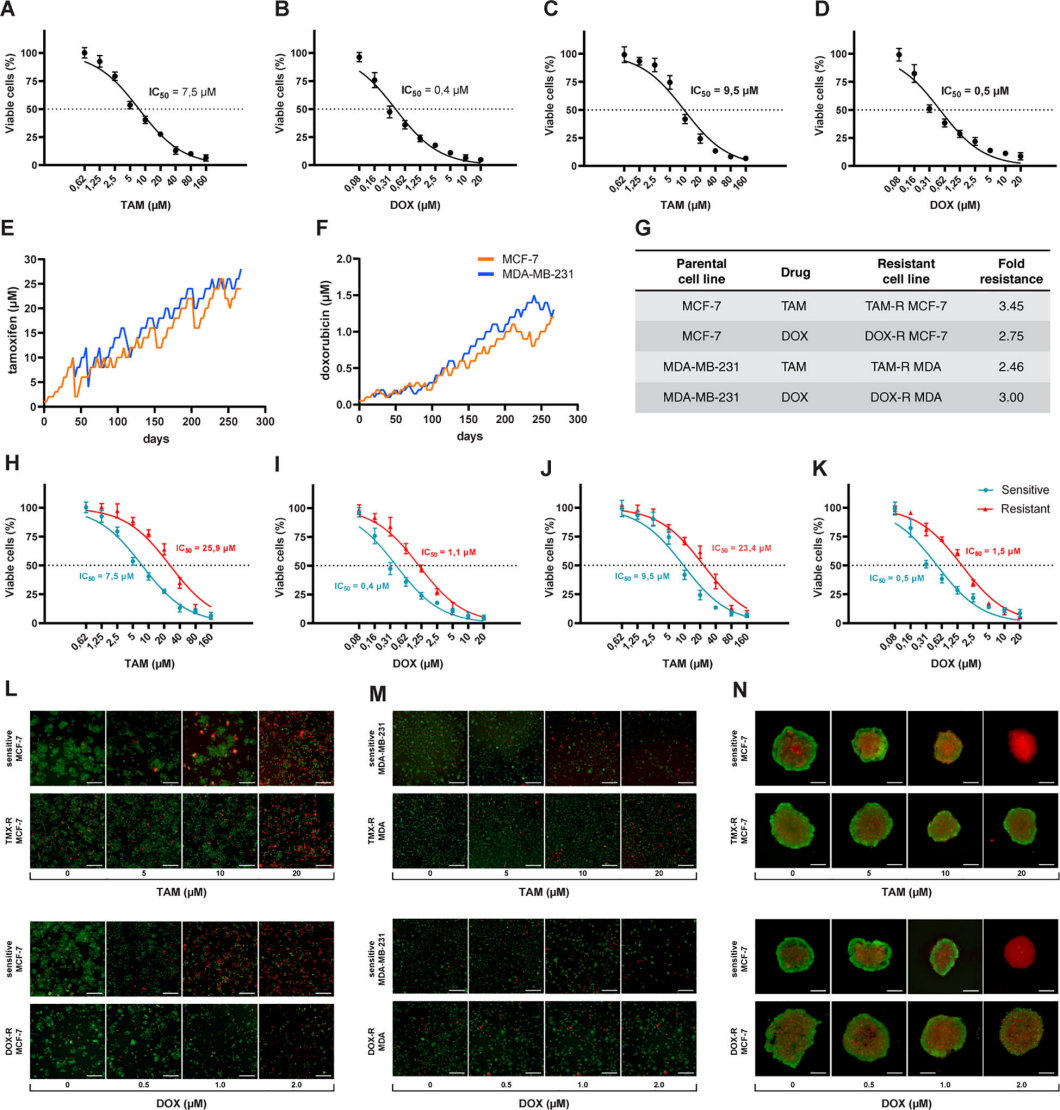
Process of generation of resistant cancer cells. Initial cell viability assay by resazurin reduction and half maximal inhibitory concentration (IC50) of sensitive MCF-7 cells treated with **(A)** tamoxifen (TAM) and **(B)** doxorubicin (DOX) and of MDA-MB-231 treated with **(C)** TAM and **(D)** DOX. Sensitive cells of MCF-7 and MDA-MB-231 exposed to **(E)** TAM and **(F)** DOX over time to generate resistant cell lines. The resistance fold calculated for each resistant cell line **(G)**, indicating the level of resistance for each drug. The IC50s of **(H)** TAM- and **(I)** DOX-resistant MCF-7 and **(J)** TAM- and **(K)** DOX-resistant MDA-MB-231 cells calculated (red line) and compared to sensitive cells (blue line). The IC50s calculated by a nonlinear regression with an adjusted confidence level of 95%. The visualization of TAM- and DOX-resistant **(L)** MCF-7 and **(M)** MDA-MB-231 cells in a two-dimensional *in vitro* setup, and **(N)** in three-dimensional spheroids of sensitive and resistant MCF-7 cells.

The first step was to calculate the IC50 values for sensitive cells exposed to a wide range of concentrations of TAM and DOX for 72 h. We estimated the IC50 of TAM and DOX in MCF-7 and MDA-MB-231 cells using a cell viability assay based on resazurin reduction. Nonlinear regression analysis showed that MCF-7 cells had lower TAM and DOX IC50s when compared to MDA-MB-231 cells (**Figures 1A–D**). The generation of drug-resistant breast cancer cell lines capable of overcoming and proliferating under exposure to higher TAM or DOX concentrations than the previously calculated IC50 of sensitive cells took nine months (**Figures 1E, F**). After the resistant cells reached this level of survival, we evaluated the IC50 values of these cell populations using the same resazurin protocol. We observed that the IC50 of MCF-7 exposed to TAM increased from 7.5 to 25.9 μM (**Figure 1H**), and the IC50 of MCF-7 exposed to DOX increased from 0.4 to 1.1 μM (**Figure 1I**). We observed an increase in TAM and DOX IC50 in resistant MDA-MB-231 cells, and in this case, the IC50 scaled from 9.5 to 23.4 μM in TAM-exposed cells (**Figure 1J**) and from 0.5 to 1.5 μM in DOX-exposed MDA-MB-231 cells (**Figure 1K**). TAM-resistant MCF-7 (TAM-R MCF-7) cells showed the highest fold resistance (3.45), followed by the sharp 3-fold resistance of DOX-resistant MDA-MB-231 cells (DOX-R MDA). DOX-resistant MCF-7 (DOX-R MCF-7) and TAM-resistant MDA-MB-231 (TAM-R MDA) cells displayed 2.75- and 2.46-fold resistance, respectively (**Figure 1G**). FDA/PI staining revealed that the resistant MCF-7 and MDA-MB-231 cell lines showed fewer PI-positive (dead) and more FDA-positive (viable) cells, even when exposed to high concentrations of TAM and DOX (**Figures 1L, M**). This dynamic was observed in 3D MCF-7 spheroids, in which sensitive cells were profoundly affected by drug treatment; however, resistant-derived spheroids showed more viable cells (**Figure 1N**).

### 3.2 Production and characterization of EVs derived from resistant cell lines

**Figure 2:**
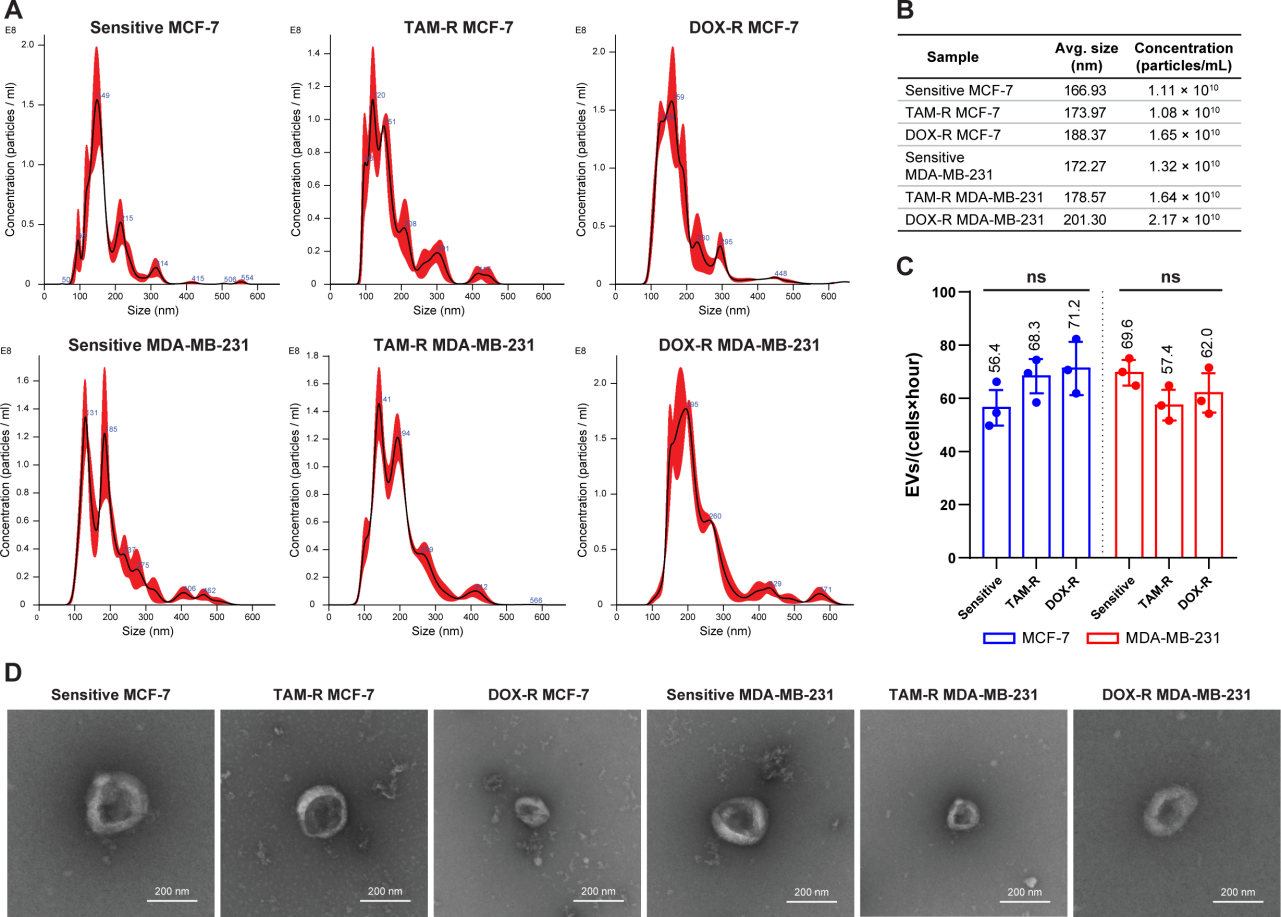
Evaluation of the production and characterization of extracellular vesicles derived from breast cancer cells. **(A)** Nanoparticle-tracking analysis showing the size distribution of extracellular vesicles (EVs) produced by sensitive and resistant MCF-7 and MDA-MB-231 cells treated with tamoxifen (TAM) and doxorubicin (DOX). **(B)** Additional information about the average size and concentration of EVs per mL. **(C)** Histogram showing the EV production of each cancer cell per hour. **(D)** Transmission electron microscopy images of sensitive and resistant EVs from breast cancer cell lines. TAM-R, tamoxifen-resistant; DOX-R, doxorubicin-resistant; ns, non-significant. The bars represent the mean with the standard deviation from three independent experiments.

The overall production of EVs was tracked using the supernatant from each cell line and the cells were plated for 24 h. The NTA results showed that sensitive and resistant cell populations produced approximately the same amount of EVs, averaging between 1.08 and 2.17 × 10^10^ EVs per mL (**Figure 2A**). Additionally, the average EV size in the parental and resistant cell lines ranged from 167 to 200 nm (**Figure 2B**). The TEM observation revealed the classic round “cup-shaped” morphology of parental and resistant breast cancer-derived EVs (**Figure 2D**). Furthermore, there were no differences in EV production between sensitive and resistant cells, and between cell lines. Each sensitive and resistant breast cancer cell line produced approximately 60 EVs per hour over 24 h (**Figure 2C**). Collectively, these results showed that there were no differences in the concentration, production, size, or shape between sensitive- and resistant-derived EVs.

### 3.3 Sensitive cells internalize drug-resistant derived EVs and resist drug treatment

**Figure 3:**
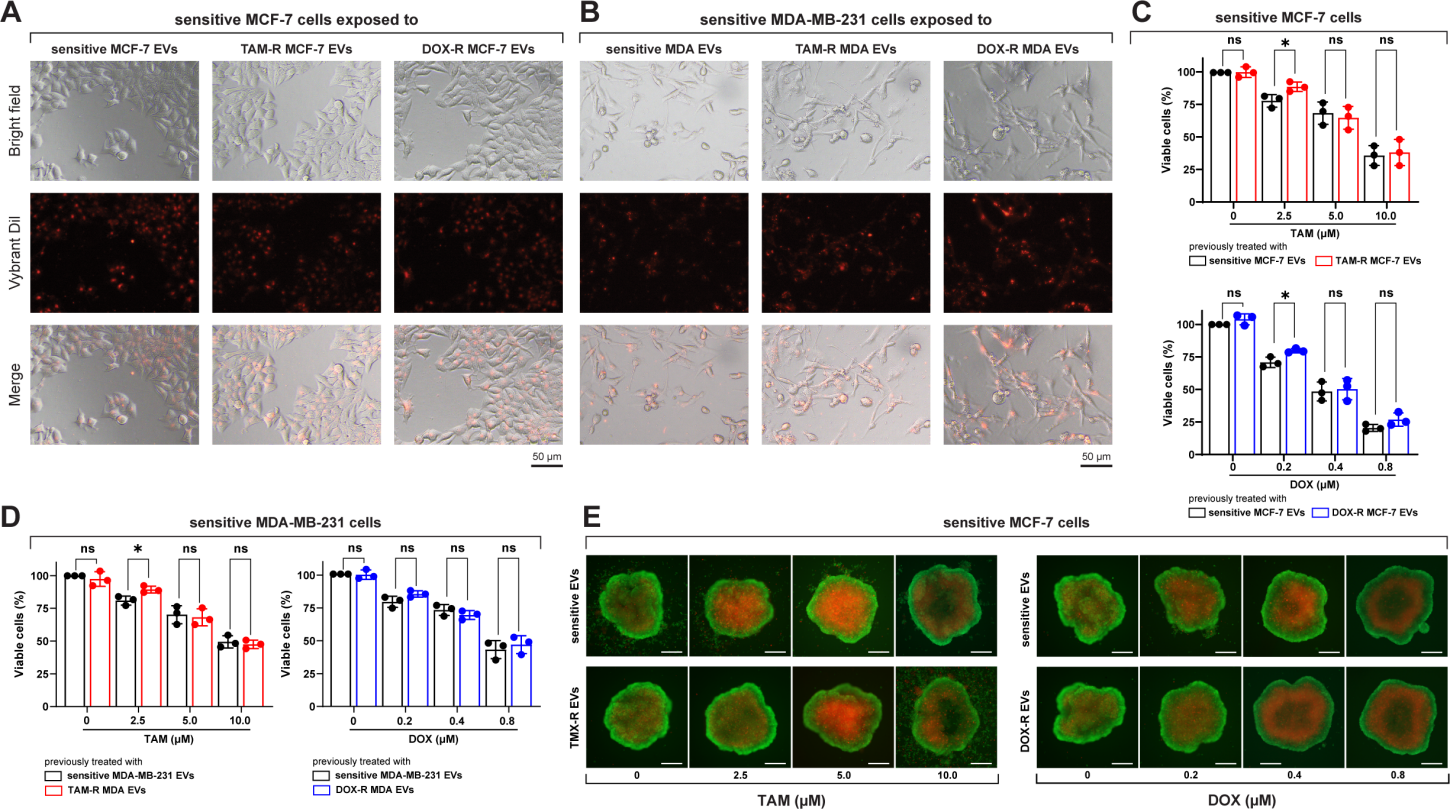
Sensitive breast cancer cells uptake resistant extracellular vesicles (EVs) and show increased cell viability. **(A)** Sensitive MCF-7 cells (Bright field) internalizing the EVs (Vybrant dil) after 24 h of exposure. **(B)** Sensitive MDA-MB-231 cells (Bright field) internalizing the EVs (Vybrant dil) after 24 h of exposure. TAM-R: tamoxifen-resistant; DOX-R: doxorubicin-resistant; ns: non-significant. A resazurin reduction assay evaluated the cell viability of sensitive **(C)** MCF-7 and **(D)** MDA-MB-231 cells exposed to resistant EVs. **(E)** Spheroids of sensitive MCF-7 cells were previously exposed to sensitive, TAM- and DOX-resistant EVs for 24 h and treated with TAM or DOX for 72h. The asterisks indicate a significant difference (p < 0.05) between tamoxifen or doxorubicin treatments when compared to no treatment (unpaired t-test).

After incubating sensitive breast cancer cells with fluorescently labeled EVs from parental and resistant cell lines, a fluorescent signal was observed inside the cells, indicating that these sensitive cells could take up TAM-R and DOX-r EVs (**Figures 3A, B**). Sensitive MCF-7 and MDA-MB-231 cells were exposed to TAM- and DOX-resistant EVs for 24 h, and subsequently treated with TAM or DOX for another 72 h. In this experiment, our results showed that the resistant-derived EVs preexposure increased the viability of sensitive cells in three different scenarios: the first and second are MCF-7 treated with 2.5 μM of TAM and 0.2 μM of DOX, and the last one is MDA-MB-231 with an increased number of viable cells after the resistant EVs preexposure and treatment with 2.5 μM of TAM (**Figure 3C**). Under our experimental conditions, high concentrations of TAM and DOX (near the original IC50 or above) showed no differences between sensitive cells exposed to sensitive or resistant EVs (**Figure 3D**). We generated MCF-7 spheroids and exposed them to sensitive or resistant EVs, which were then treated with TAM or DOX. In the images, more dead cells (red labeled) can be observed in the spheroid previously exposed to sensitive EVs and treated with 2.5 μM of TAM than the one previously exposed to TAM-R EVs. Moreover, the spheroid that received 0.2 μM of DOX and was previously exposed to sensitive EVs showed more dead cells than the one exposed to DOX-R EVs (**Figure 3E**).

### 3.4 Transcriptome-wide analysis of drug resistant derived EVs influence on sensitive cells

**Figure 4:**
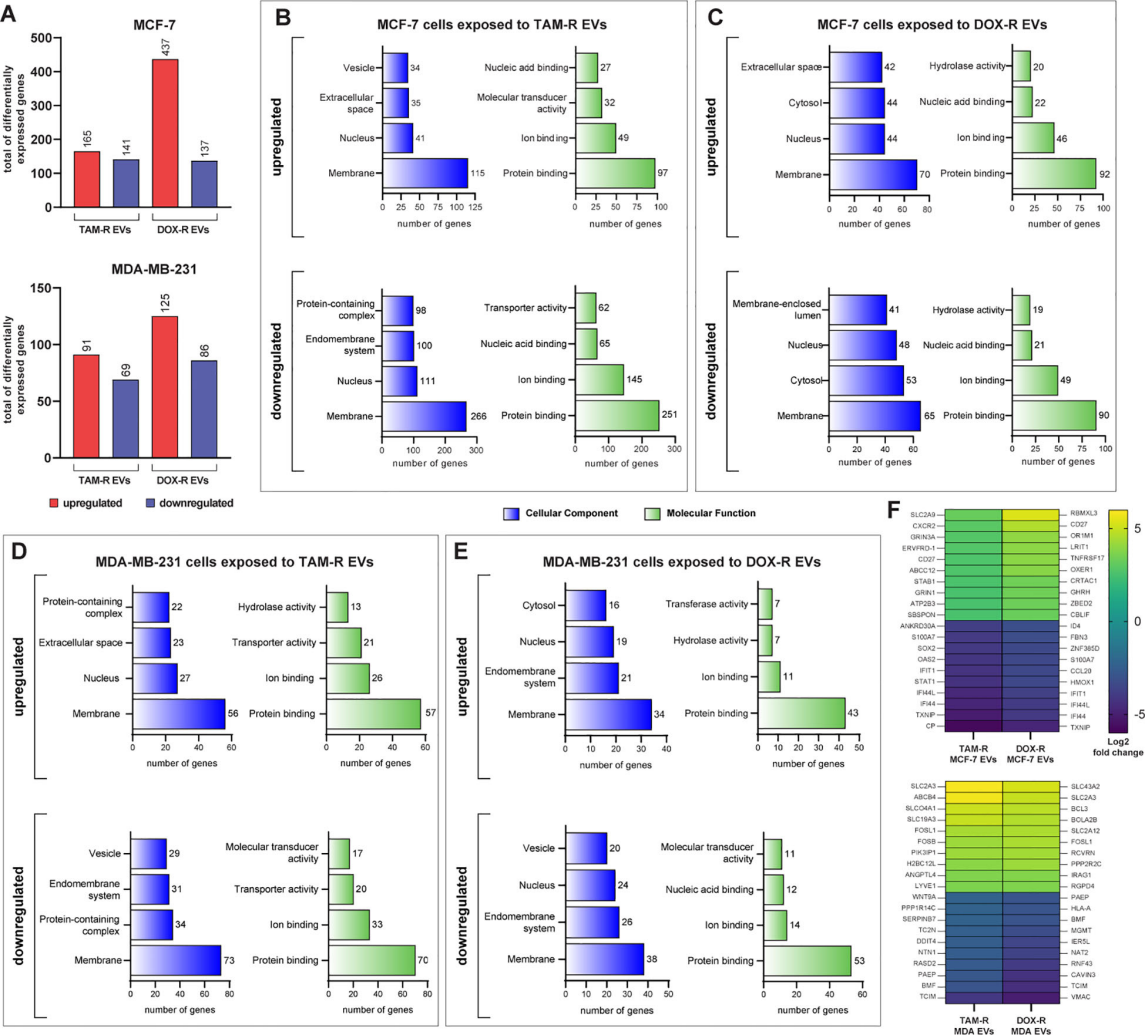
Transcriptome-wide analysis showing the differential expression in sensitive breast cancer cell lines exposed to resistant extracellular vesicles (EVs) for 24 hours. **(A)** Histogram of the total number of differentially expressed genes (DEGs) in MCF-7 and MDA-MB-231. Distribution of gene ontology (GO) slim terms associated with MCF-7 transcripts after tamoxifen-resistant EVs (TAM-R EVs) exposure **(B)** and doxorubicin-resistant EVs (DOX-R EVs) exposure **(C)**. For MDA-MB-231, the distributions of GO-slim enriched terms after TAM-R and DOX-R EV exposure are shown in **(D, E)**, respectively. **(F)** Heatmap showing each condition’s top 10 up- and downregulated genes based on their log2 fold change.

To better understand the influence of drug-resistant EVs on the sensitive MCF-7 and MDA-MB-231 transcriptomes, we performed RNA sequencing (RNA-Seq) analysis in these two cell lines after exposure to TAM-R and DOX-R EVs for 24 h. After sequencing and mapping, 306 genes were considered DEGs of MCF-7 cells exposed to TAM-R EVs, of which 165 were upregulated and 141 were downregulated, and 574 genes were labeled as DEGs of MCF-7 cells exposed to DOX-EVs, separated into 437 upregulated and 137 downregulated genes, using the previously explained cutoff filtering (**Figure 4A**). In MDA-MB-231 cells exposed to TAM-R, we identified 160 DEGs, of which 91 were upregulated and 69 were downregulated. Additionally, 211 genes were identified as DEGs and were separated into 125 upregulated and 86 downregulated genes (**Figure 4A**). We analyzed the relationships to cellular components, and molecular functions using GO slim subsets to obtain a comprehensive overview of the DEG lists. This broad analysis is the first step in the transcriptome analysis pipeline, giving general information about which locations, mechanisms or cellular functions have more DEGs in breast cancer cells exposed to EVs derived from resistant cells for further investigation. This GO analysis revealed that the most enriched term for all conditions in which MCF-7 cells were exposed to resistant EVs was related to the membrane, followed by the “nucleus” term. Additionally, in the Molecular Function category, the most enriched term in MCF-7 cells exposed to resistant EVs was related to protein binding (**Figures 4B, C**). We observed the same pattern of enriched GO terms in the DEG list of MDA-MB-231 cells exposed to TAM-R- or DOX-R-derived EVs (**Figures 4D, E**). We generated a heatmap containing the top 10 most upregulated and downregulated genes under each resistant EV exposure condition (**Figure 4F**). Inside this narrow list, we observed the upregulation of DEGs related to solute transport (solute carrier family SLC), ABC transporters (*ABCC12* and *ABCB4*), tumor growth transcription factors (*FOSL1* and *FOSB*), and the downregulation of genes induced by interferon (*IFI44L*, *IFI44*, and *IFIT1*). In addition, the tumor suppressors STAT1 and human leukocyte antigen class I (*HLA-A*) were downregulated.

**Figure 5:**
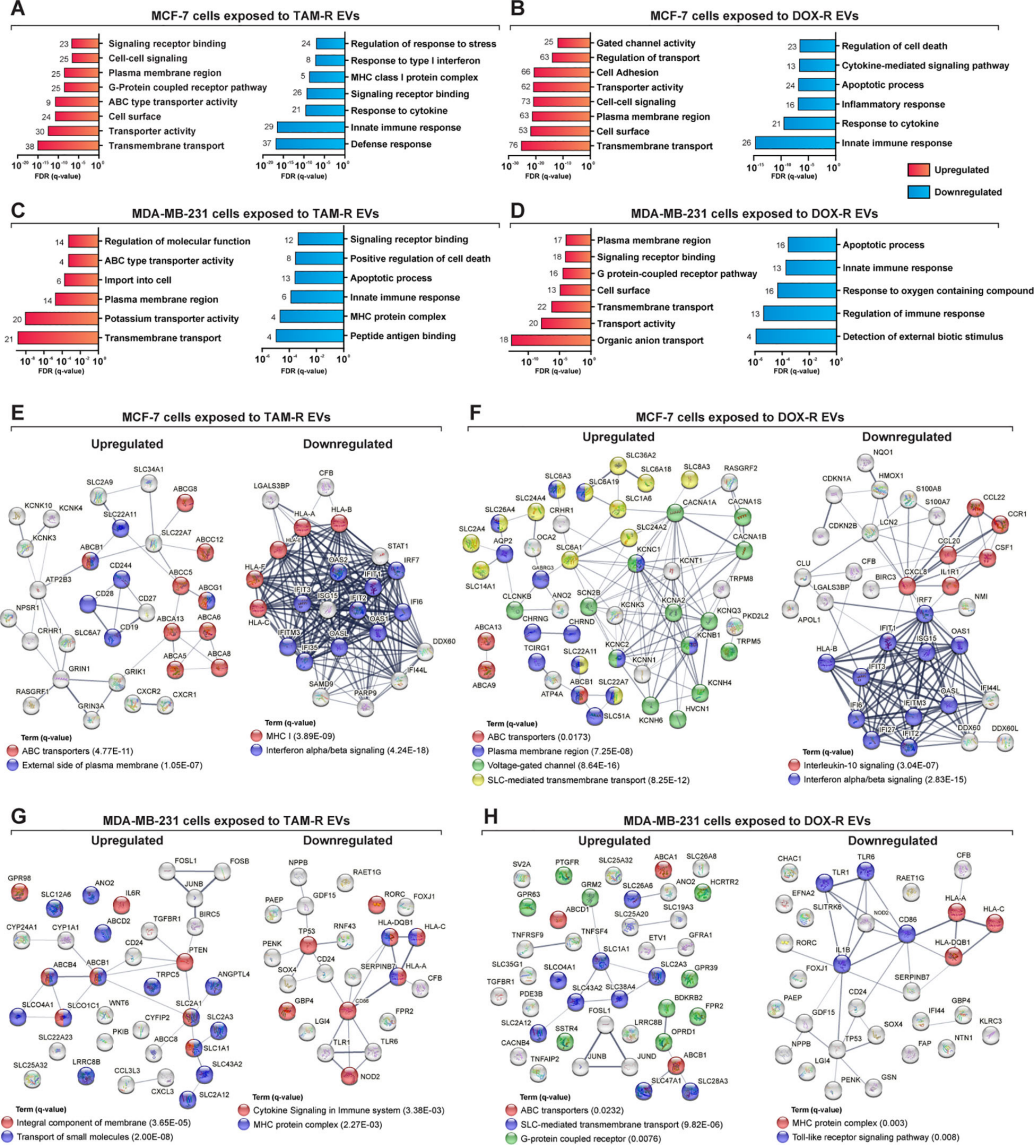
Deep over-representation analysis of the differentially expressed genes (DEGs) in breast cancer cells exposed to extracellular vesicles (EVs) released by tamoxifen-resistant (TAM-R) and doxorubicin (DOX-R) cell lines. The gene ontology (GO) analysis enriched more specific terms considering the up- and downregulated DEGs in sensitive MCF-7 cells exposed to **(A)** TAM-R EVs and **(B)** DOX-R EVs for 24 h For sensitive MDA-MB-231 cells, the enriched terms for up- and downregulated DEGs after exposure to TAM-R and DOX-R EVs are displayed in **(C, D)**, respectively. The number in front of each bar represents the total of enriched DEGs for each term. The x-axis of A to D represents the adjusted *p*-value (*q*-value) with a false discovery rate of 1%. Protein-protein interaction networks were generated using the DEG lists as references. **(E, F)** display the networks created by the MCF-7 DEGs after exposure to TAM-R and DOX-R EVs, respectively. For MDA-MB-231 cells, the exposure of TAM-R and DOX-R EVs generated the networks represented in **(G, H)**, respectively.

To further understand the biological function of the DEGs, we performed a more in-depth GO analysis using the GSEA over-representation parameter with a 1% FDR significance level, and functionally related gene sets were enriched. The upregulated DEGs in MCF-7 cells exposed to TAM-R and DOX-R EVs were related to transport activity in the plasma membrane region via ABC transporters, gated channel activity, and G protein-coupled receptors (**Figures 5A, B**). The downregulated DEGs of MCF-7 cells exposed to TAM-R enriched terms associated with innate immune response and MHC I activity, especially the response to type I interferon, while the downregulated DEGs of MCF-7 cells exposed to DOX-R enriched terms associated with immune response. However, this list enriched terms related to cell death processes, including apoptosis (**Figures 5A, B**). The DEGs from MDA-MB-231 cells exposed to TAM-R and DOX-R EVs followed the same pattern as in MCF-7 cells; the upregulated genes were enriched in terms of the transport of molecules via ABC transporters, G-protein receptors, and potassium ion transmembrane activity on the cell surface. In contrast, the downregulated DEGs of MDA-MB-231 cells exposed to both TAM-R and DOX-R EVs showed enrichment of terms related to cell death, apoptotic processes, and innate immune responses (**Figures 5C, D**).

ABC transporters are involved in acquired resistance in cancer cells. Using the DEGs lists in STRING to generate physical and biological interaction networks between the proteins encoded by these genes, we observed many upregulated ABC transporters in both cell lines after exposure to resistant EVs, including *ABCB1* (P-glycoprotein); several ABC1 transporters (*ABCA1*, *ABCA5*, *ABCA6*, *ABCA8*, *ABCA9*, *ABCA13*, and *ABCB5*); and multidrug resistance-associated proteins (MRPs: *ABCC5* and *ABCC12*), especially in cells exposed to TAM-R EVs (**Figures 5E, G**). Our results indicate the upregulation of genes is directly related to the transport of molecules across membranes. We observed SLC genes in all upregulated DEG, especially in cancer cells exposed to DOX-R EVs (**Figures 5F, H**). Additionally, the networks showed the upregulation of genes associated with voltage-gated channels in MCF-7 cells that received DOX-R EVs, and the upregulation of genes related to the G-protein coupled receptor in MDA-MB-231 cells that were exposed to DOX-R EVs. When looking at the downregulated networks, we can observe the reduced expression of interferon-induced proteins, such as *IFIT1*, *IFIT2*, and *IFIT3*, in MCF-7 cells exposed to TAM-R and DOX-R EVs. The MHC protein complex was enriched in all the downregulated lists, in which we observed the downregulation of several HLA genes, including *HLA-A*, *HLA-B*, *HLA-C*, *HLA-E*, and *HLA-F*.

### 3.5 Resistant EVs modulate drug resistance-related genes and increase the survivability of cancer cells

**Figure 6:**
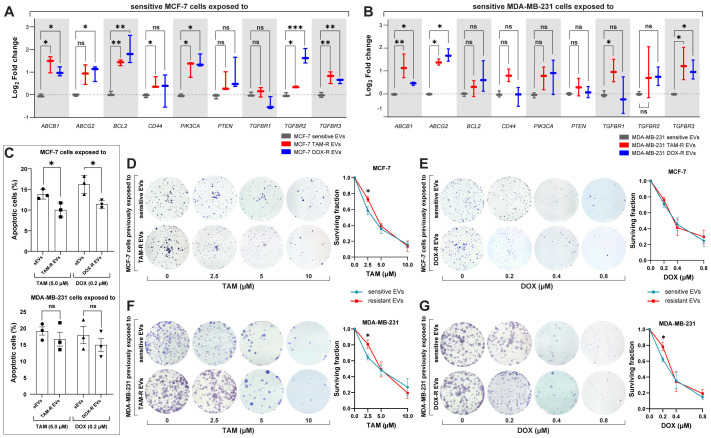
Resistant extracellular vesicles (EVs) induce the upregulation of genes associated with acquired drug resistance and increase the sensitive cells’ survivability. The quantification of several genes by quantitative polymerase chain reaction (qPCR) of **(A)** sensitive MCF-7 exposed to tamoxifen-resistant (TAM-R) and doxorubicin-resistant (DOX-R) EVs. **(B)** The gene expression level of sensitive MDA-MB-231 cells exposed to TAM-R and DOX-R. **(C)** The apoptotic rate by annexin V/propidium iodide labeling in sensitive MCF-7 and sensitive MDA-MB-231 previously exposed to resistant EVs and treated with TAM and DOX. **(D)** Representative images and the clonogenic surviving fraction of MCF-7 cells previously exposed to TAM-R and **(E)** DOX-R EVs treated with the respective drug. **(F)** Representative images and the clonogenic surviving fraction of MDA-MB-231 cells previously exposed to TAM-R and **(G)** DOX-R EVs treated with the respective drug. Values are displayed as the mean ± standard deviation from three independent experiments. The asterisks indicate a significant difference between TAM-R EVs or DOX-R EVs exposure compared to sensitive EV effects (unpaired *t*-test). *p < 0.05, **p < 0.01, ***p < 0.001, ns, not significant.

According to our transcriptome results, resistant EVs influence several cellular mechanisms, particularly the transport of molecules and cell death-/survival-related functions. To further explore these findings, we analyzed the effects of resistant EVs on the expression of significant driver genes related to drug resistance and cell survival. The qPCR results showed that resistant EVs upregulate the expression of ABC transporters (*ABCB1* and *ABCG2*) and transforming growth factor-β receptor genes (*TGFBR1*, *TGFBR2*, and *TGFBR3*) in both sensitive cell lines, except for MCF-7 cells exposed to resistant EVs, which showed no difference in *TGFBR1* expression in comparison with sensitive EVs exposure (**Figures 6A, B**). We observed the upregulation of *PIK3CA* and downregulation of *BCL2* in MCF-7 cells after exposure to resistant EVs, which is an interesting result that is directly related to apoptosis. The downregulation of genes associated with apoptosis suggests that resistant EVs may inhibit this cell death mechanism. To further explore this finding, we analyzed the effects of TAM- and DOX-R EVs on sensitive cells using a cellular assay for annexin V labeling. After 24 h of initial exposure to resistant EVs, both sensitive cell lines were treated with TAM or DOX for 72 h. The results showed that TAM-R and DOX-R EVs reduced the population of apoptotic MCF-7 cells (**Figure 6C**). In contrast, we observed neither a difference in *PIK3CA* and *BCL2* expression nor in the apoptosis rate in MDA-MB-231 cells exposed to resistant EVs compared to the counterpart-sensitive EVs.

Taking the upregulation of transforming growth factor-β- (TGF-β) related genes as a reference, we investigate the cytostatic effects of resistant EVs to ascertain whether this upregulation of proliferation-related genes translates into increased cell growth and survivability. A clonogenic assay was performed, and the results showed that resistant EVs increased the survival rate and colony formation in the long term. We observed that MCF-7 TAM-R EVs increased the survivability and proliferation of sensitive cells after TAM treatment (2.5 μM) when compared to MCF-7 that received sensitive EVs and the same TAM treatment (**Figure 6D**); however, we did not find any difference in MCF-7 exposed to DOX-R EVs (**Figure 6E**). MDA-MB-231 cells previously exposed to TAM-R EVs showed a significant increase in colony formation after being treated with 2.5 μM of TAM compared to the cells that received sensitive EVs (**Figure 6F**). Additionally, MDA-MB-231 exposed to DOX-R EVs showed a higher survival fraction when treated with 0.2 μM of DOX compared to cells that received sensitive EVs (**Figure 6G**).

### 3.6 Resistant EVs modulate the macrophage’s cytokine release profile

**Figure 7:**
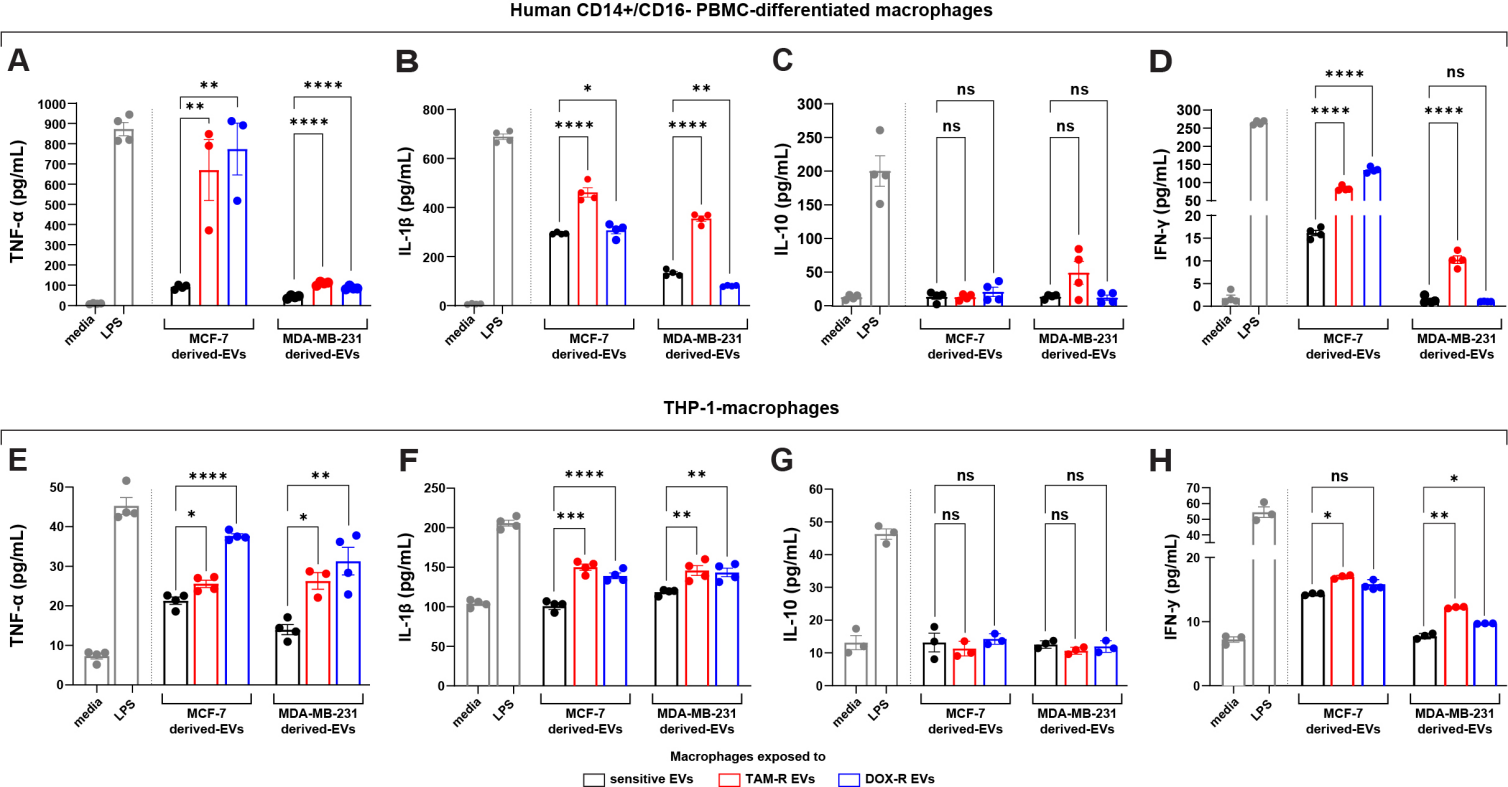
Extracellular vesicles (EVs) released by tamoxifen-resistant (TAM-R) and doxorubicin-resistant (DOX-R) breast cancer cell lines modulate the cytokine production and release of human macrophages. Human CD14+/CD16- peripheral blood mononuclear cells (PBMC)-derived macrophages were exposed to EVs released by resistant MCF-7 and MDA-MB-231, and the quantification level of **(A)** tumor necrosis factor (TNF)-α, **(B)** interleukin (IL)-1β, **(C)** IL-10, and **(D)** interferon (IFN)-γ were evaluated. Additionally, THP-1-macrophages were exposed to resistant EVs, and the secretion levels of **(E)** TNF-α, **(F)** IL-1β, **(G)** IL-10, and **(H)** IFN-γ measured by the ELISA assay. Values are displayed as the mean ± standard deviation from three independent experiments. The asterisks indicate a significant difference between TAM-R EVs or DOX-R EVs exposure compared to sensitive EV effects (unpaired *t*-test). *p < 0.05, **p < 0.01, ***p < 0.001, ****p < 0.0001; ns, not significant; media, RPMI 1640; LPS, lipopolysaccharide (positive control).

Given that genes directly related to the immune response were modulated by resistant EVs in both breast cancer cell lines, we performed ELISA to detect specific cytokines released by different human macrophages when exposed to TAM-R and DOX-R EVs from MCF-7 and MDA-MB-231 resistant cell lines. The human PBMC macrophages were exposed for 24 h to resistant EVs, and then we collected the supernatant and evaluated the levels of several cytokines. In this experiment, we observed increased levels of TNF-α and IL-1β after all resistant EVs exposure (**Figures 7A, B**); we observed an increase in IFN-γ levels after these macrophages were exposed to TAM-R EVs from the TAM-R MDA cell line (**Figure 7C**); however, we did not find any difference between the sensitive and resistant EVs in the IL-10 levels in all tested conditions (**Figure 7D**). Additionally, using the human monocyte cell line THP-1 transformed into macrophages by PMA, we further analyzed the impact of resistant EVs on macrophage cytokine release patterns. We observed results similar to those obtained for PBMC. We observed increased levels of TNF-α and IL-1β (**Figures 7E, F**) and no differences in IL-10 levels (**Figure 7G**) in all tested conditions. The difference lies in IFN-γ level measurement. We observed a significant increase in IFN-γ concentration in the supernatant from macrophages exposed to TAM-R and DOX-R EVs from MCF-R and TAM-R EVs from MDA-R cell lines (**Figure 7H**).

## 4 Discussion

The two breast cancer cell lines, MCF-7 and MDA-MB-231, were selected due to their representation of distinct phenotypes and genotypes of the disease. MCF-7 cells are hormone-dependent, being positive for estrogen and progesterone receptors. This characteristic makes them more sensitive to drugs specifically designed to target hormone receptors, such as TAM, an estrogen receptor modulator. Additionally, MCF-7 cells exhibit a luminal/epithelial phenotype and form spontaneous spheroids *in vitro*. In contrast, MDA-MB-231 cells are triple negative, which contributes to their increased resistance to various drug treatments. This lack of response, combined with their mesenchymal phenotype, makes MDA-MB-231 cells more aggressive and invasive compared to MCF-7 cells (30). Given that both cell lines have demonstrated multidrug resistance and exhibit differential susceptibilities during the development of acquired drug resistance, investigating the effects of extracellular vesicle on MCF-7 and MDA-MB-231 cells in a single study encompasses multiple aspects of cancer progression.

Several large-scale studies have been performed to investigate the role of EVs in breast cancer progression, particularly in the acquisition of a drug-resistant phenotype during chemotherapy (31), including quantitative proteomics (32), epigenetic profiling (33), and miRNA expression profiles (34). Our contributions include the generation of high-level laboratory-resistant breast cancer cell lines from parental MCF-7 and MDA-MB-231 cells, followed by transcriptome-wide analysis combined with different cellular endpoints. These findings might help understand the influence of EVs from resistant breast cancer cells in sensitive cells to trigger acquired drug resistance. Initially, the generation of resistant cell lines following the guidelines of McDermott (14) resulted in a reliable tool for studying drug resistance *in vitro*. The comparison of IC50 and the resistance fold showed a disparity in resistance levels between sensitive and resistant cells. Other recent studies with TAM-resistant MCF-7 and TAM-resistant MDA-MB-231 showed similar IC50 values to our results (35, 36). Additionally, DOX-resistant MCF-7 and MDA-MB-231 cells showed an IC50 range of 0.9 to 1.5 μM, which is close to what we observed in our DOX-resistant cell lines (37, 38).

EVs are highly heterogeneous in terms of composition; however, their morphology follows the same spherical pattern (39). More studies are needed on the EV production rate per cell. According to Chiu et al. (40), MCF-7 and MDA-MB-231 cells generated approximately 60 EVs per hour. These results are similar to ours, even though the resistant cell lines did not change their EV secretion rate. Our data suggest no differences in the concentration, production, or characterization of sensitive and resistant EVs derived from breast cancer cells. The interaction between EVs and cells and the internalization of EVs are much less studied than EV formation and secretion, and information concerning whether EVs must be internalized to trigger a few effects in the cell membrane is rare (41). We observed that resistant EVs interact with and are internalized by sensitive cells in a 24-h time frame with no cytotoxic effects. Consistent with our results, EVs released by SW480 human colorectal cancer cells were internalized by THP-1 macrophages; however, SW480-derived EVs showed minor cytotoxicity towards these macrophages (42).

Considering the ability of our resistant cells to survive TAM or DOX treatment and the literature reports on the transfer of drug resistance via EVs, we hypothesized that EVs released by resistant cells may increase the number of viable cells after drug treatment in sensitive cells that have never been exposed to TAM or DOX by providing some form of drug resistance to these cells. We observed that sensitive breast cancer cells previously exposed to resistant EVs showed higher viability than cells exposed to sensitive EVs in 2D culture with MCF-7 and MDA-MB-231 cells and in 3D culture with MCF-7 spheroids. Based on these results, RNA sequencing was selected as the platform for further investigation of resistant EVs and their roles in acquired drug resistance. Bioinformatics analysis showed that resistant EVs from MCF-7-R and MDA-R cells influence transport-related mechanisms, cell death, and the innate immune response. The most upregulated DEGs in MCF-7 and MDA-MB-231 cells were associated with transport activity.

Several studies have revealed the direct participation of transporters in drug resistance acquisition, especially ABC transporters, specifically P-glycoprotein (ABCB1). Studies have suggested that P-gp can be directly transferred to sensitive cells via EVs, or that its expression can be increased by transferring miRNAs from resistant cells to sensitive cells (43). Our results revealed an upregulation of *ABCB1*, responsible for P-gp encoding, in MCF-7 and MDA-MB-231 cells exposed to TMX-R and DOX-R EVs, suggesting that the resistant EVs in our conditions increased the expression of P-gp as a general drug resistance mechanism, independent of the cell line or drug type. This non-specific activity and transfer of ABCB1, regardless of whether the drug is a direct substrate of ABCB1 efflux transport, was observed in EVs released by KBv200 cells overexpressing ABCB1. These EVs increase ABCB1 expression in sensitive cells after treatment with drugs with different therapeutic mechanisms (44). In the context of breast cancer, there are few reports available on the influence of P-gp via EVs on drug resistance; for example, multidrug-resistant MCF-7 cells specifically transfer P-gp to EVs, which mediate resistance to several anticancer drugs (45). However, to date, there is no information on ABCB1 transfer via EV in MDA-MB-231 cells during the acquisition of drug resistance. We observed the upregulation of the *ABCG2* gene, which encodes breast cancer resistance protein (BCRP), in sensitive cells exposed to resistant EVs. Other studies have shown the accumulation of ABCG2 inside EVs from resistant cell lines (46), which induces drug resistance in sensitive cells (47). Besides P-gp and BCRP, our results showed an upregulation of several genes that are directly associated with acquired drug resistance during chemotherapy, frequently called multidrug resistance (MDR) transporters, including *ABCB4*, *ABCC5*, and *ABCG1*, to name a few. EVs containing these MDR transporters can induce a pump efflux from the cytoplasm to extracellular space in sensitive breast cancer cells (48, 49). Other studies have shown that MDR transporters contribute to chemotherapy resistance; however, do not participate in the acquired drug resistance process (50, 51). Our findings are the first transcriptome-wide study showing the influence of MDR transporters modulated by resistant EVs in sensitive breast cancer cells.

Membrane transporters are crucial for the acquisition of drug resistance, and we observed the upregulation of genes associated with SLC-mediated transmembrane transport, voltage-gated channels, and G-protein coupled receptors. While ABC transporters are active and dependent on ATP hydrolysis, SLC transporters act as secondary active transporters, using an ion gradient to handle diverse substrates (52). SLC transporters play important roles in the development of drug resistance. A recent study showed that these transporters are highly expressed in patients resistant to chemotherapy (53). In combination with high expression of P-gp, SLC transporters enhance cell proliferation in human leukemia, facilitating the acquisition of drug resistance (54). Additionally, voltage-gated channels, especially calcium and potassium channels, are often reported to be overexpressed in different types of cancer, including breast cancer, where they induce cell proliferation, tumor growth, and drug resistance (55, 56). In malignant cells, calcium mobilization and the activation of calcium signaling are directly related to EV biogenesis, facilitating plasma membrane vesiculation (57); hence, it may act as a secondary drug resistance process. Furthermore, abnormal expression of potassium voltage-gated channels contributes to breast cancer progression and drug resistance (58, 59); however, the interaction between potassium channels and EVs is unclear. Potassium channels are activated by G protein-coupled receptors (GPRs), which play an essential role in many cellular processes. In the context of cancer, they are overexpressed and associated with tumor growth and metastasis (60), and more recently, GPRs have been reported as promoters of acquired drug resistance to different drugs in breast cancer cell lines (61, 62). Our results showed the upregulation of several types of membrane transporters, including drug resistance drivers, such as *ABCB1* and *ABCG2*, in combination with SLC transporters, voltage-gated channels, and GPRs, revealing that EVs may carry a powerful setup to induce drug resistance with multiple approaches in adjacent or distant cancer cells. However, a few aspects of this puzzle are still unknown, including acquired drug resistance induced by EVs released by resistant cancer cells.

On the other side of the spectrum, we observed that many genes associated with cell death, especially apoptosis, were downregulated after exposure to resistant EVs, which was expected after the increase in viability of sensitive cells exposed to resistant EVs. Inhibition of apoptotic signaling pathways such as the p53 pathway is directly related to cell survival, proliferation, and drug resistance; the upregulation of pro-apoptotic regulators promotes drug resistance (63). We observed the upregulation of anti-apoptotic factors, such as BCL-2 and PIK3CA, and the downregulation of *TP53* (responsible for encoding p53) in MCF-7 cells after exposure to resistant EVs. These findings correlated with the decrease in the apoptotic cell population in sensitive MCF-7 cells previously exposed to resistant EVs and after treatment with TAM and DOX. Recently, EVs derived from cancer cells were shown to reduce the apoptosis rate via upregulation of *BCL2* in other cancer cells (64), and tumor-derived EVs containing extracellular matrix molecules induced mutations in *PIK3CA*, which promoted proliferation and invasion in breast cancer (65). Despite the enrichment of terms related to cell death, we did not observe changes in the apoptotic population of sensitive MDA-MB-231 cells exposed to resistant EVs and treated with TAM and DOX. Additionally, BCL-2 and PIK3CA levels showed no significant differences. Further investigation is needed to understand the anti-apoptotic events displayed in RNA-Seq, which were not observed in other experiments. Our results, especially in MCF-7 cells, showed that EVs released by resistant breast cancer cell lines can modulate the apoptotic process via the upregulation of anti-apoptotic regulators and the downregulation of pro-apoptotic factors, inhibiting cell death and therefore promoting drug resistance in sensitive cancer cells. However, the exact mechanism by which these transcriptional changes occur remains unclear. It may be related to the transfer of miRNAs, as suggested by other authors (66, 67).

Another feature that cancer cells exhibit to evade apoptosis, survive, proliferate, and resist chemotherapy is related to TGF-β signaling, and many studies showed that miRNA and proteins associated with the TGF-β pathway, including TGF-β receptors (TGFBR), were observed in cancer-derived EVs (68). The ability of EVs to transfer TGFBR was observed in breast cancer, in which these cancer EVs transferred activated TGFBRs to CD8+ lymphocytes, exhausting them and transferring TGFBRs to MDA-MB-231 cells, promoting metastasis, paclitaxel, and DOX resistance (69). Similar results have been reported using TGFBR-rich EVs from MCF-7 cells, which induce DOX resistance. According to these studies and the upregulation of TGFBRs, we observed in sensitive cancer cells after being exposed to resistant EVs, the TGFBR transfer is crucial for cancer cells to survive and develop drug resistance in sensitive cells. Consistently, this survival mechanism supported by TGF-β signaling via EVs is linked to the clonogenic surviving fraction by the number of colonies that we observed in MDA-MB-231 cells previously exposed to TAM-R EVs and MCF-7 cells exposed to TAM-R and DOX-R EVs. In this case, resistant EV exposure was able to increase the survival rate of sensitive cells only at the lowest tested concentration, suggesting that the acquired resistance induced by EVs increases the survivability of sensitive cells. However, it cannot overcome the drug effect of high concentrations after a period without any stimuli with resistant EVs.

In the context of breast tumor, cancer cells influence the role of macrophages, which are the most abundant immune cells within the tumor microenvironment, depending on the stage of breast cancer progression (70). Tumor-associated macrophages in breast cancer exhibit a range of functions and phenotypes. Consequently, specific subsets of macrophages perform distinct and varied roles, either modulating tumor progression or exerting anticancer effects (71, 72). The downregulation of genes associated with innate immune responses observed in the RNA-seq data prompted us to investigate the influence of resistant EVs on macrophages. Cancer-derived EVs and their effects on macrophages are popular topics in cancer biology. Simultaneously, many studies reported that EVs released by cancer cells promote an anti-inflammatory phenotype polarization in macrophages as part of the immune system evasion hallmark during tumor progression, which is frequently associated with poor survival outcomes (73, 74). In the broader context of breast cancer biology, anti-inflammatory macrophages play a crucial role in the development of drug resistance by influencing various signaling pathways in cancer cells. They contribute to resistance mechanisms through the overexpression of PI3K/AKT factors, which promote tumor growth (75, 76). Several other studies have shown that cancer-derived EVs can trigger a pro-inflammatory phenotype in macrophages; consequently, this process is associated with better clinical outcomes (77, 78). When we exposed human macrophages to resistant breast cancer-derived EVs, we observed a pro-inflammatory profile with increased TNF-α, IL-1β, and IFN-γ expression, exhibiting anti-tumoral characteristics. The pro-inflammatory response involves TNF-α expression, which activates of T lymphocytes and Natural Killer cells. IFN-γ released by macrophages triggers Toll-like receptor activation and induces cell cycle arrest in cancer cells, while IL-1β acts as a significant driver of inflammation (79, 80). However, both IL-1β and TNF-α exhibit paradoxical pro-tumor effects that are associated with metastasis in breast cancer (81, 82). This potential pro-inflammatory response from macrophages contrasts with the downregulation of innate immune response-related genes in sensitive cells exposed to resistant EVs. These findings suggest that breast cancer-resistant EVs modulate immune system factors in sensitive cancer cells to evade immune detection, such as the downregulation of several MHC-related genes, including HLAs, resulting in unpaired antigen presentation. However, there is a lack of information regarding the modulation of the immune system by cancer-derived EVs in other cancer cells. Collectively, our data show that resistant EVs may play a dual role in cancer progression: they induce changes in cancer cells to evade immune surveillance, whereas these EVs may carry molecules that trigger a pro-inflammatory signature in macrophages, which represents an interesting mechanism for future immunotherapies by exploiting cancer EVs downfall.

In summary, the data discussed in this study revealed important information about EVs released by resistant breast cancer cell lines, in which initially resistant EVs reduced the toxicity of chemotherapeutic drugs, such as TAM and DOX, in sensitive MCF-7 and MDA-MB-231 cells as part of the development of acquired drug resistance. Moreover, our data highlighted that resistant EVs can modulate the transcription pattern of breast cancer cells, inducing the upregulation of transporters and downregulating immune system factors and cell death-related genes, which are the mechanisms of acquired drug resistance. Additionally, our findings shed light on a potential target for future immunotherapies related to the ability of macrophages to activate a pro-inflammatory pattern when exposed to resistant EVs. Understanding the interactions between EV released by resistant and sensitive cancer cells remains challenging. There are few such reports in the literature; however, our data provide substantial insights for future research on acquired drug resistance influenced by cancer EVs.

## Data Availability

The data presented in the study are deposited in the GEO repository, accession number GSE273705.
