# Corrigendum: Extracellular vesicles from human breast cancer-resistant cells promote acquired drug resistance and pro-inflammatory macrophage response

**DOI:** 10.3389/fimmu.2025.1595885

**Published:** 2025-04-16

**Authors:** Patrick Santos, Caroline P. Rezende, Renan Piraine, Bianca Oliveira, Francielle B. Ferreira, Vinicius S. Carvalho, Rodrigo T. Calado, Matteo Pellegrini, Fausto Almeida

**Affiliations:** ^1^ Department of Biochemistry and Immunology, Ribeirão Preto Medical School, University of São Paulo, Ribeirão Preto, SP, Brazil; ^2^ Department of Medical Imaging, Hematology, and Oncology, Ribeirão Preto Medical School, University of São Paulo, Ribeirão Preto, Brazil; ^3^ Department of Molecular, Cell, and Developmental Biology, University of California, Los Angeles, CA, United States

**Keywords:** chemoresistance, tamoxifen, doxorubicin, immunomodulation, membrane transporters

In the published article, there was an error in **Figure 6** as published. In panel A and B of **Figure 6**, the color in the graph legend corresponding to MCF-7 and MDA-MB-231 sensitive EVs should be in grey, not in blue. The corrected **Figure 6** and its caption “Figure 6. Resistant extracellular vesicles (EVs) induce the upregulation of genes associated with acquired drug resistance and increase the sensitive cells’ survivability. The quantification of several genes by quantitative polymerase chain reaction (qPCR) of (A) sensitive MCF-7 exposed to tamoxifen-resistant (TAM-R) and doxorubicin-resistant (DOX-R) EVs. (B) The gene expression level of sensitive MDA-MB-231 cells exposed to TAM-R and DOX-R. (C) The apoptotic rate by annexin V/propidium iodide labeling in sensitive MCF-7 and sensitive MDA-MB-231 previously exposed to resistant EVs and treated with TAM and DOX. (D) Representative images and the clonogenic surviving fraction of MCF-7 cells previously exposed to TAM-R and (E) DOX-R EVs treated with the respective drug. (F) Representative images and the clonogenic surviving fraction of MDA-MB-231 cells previously exposed to TAM-R and (G) DOX-R EVs treated with the respective drug. Values are displayed as the mean ± standard deviation from three independent experiments. The asterisks indicate a significant difference between TAM-R EVs or DOX-R EVs exposure compared to sensitive EV effects (unpaired *t*-test). *p < 0.05, **p < 0.01, ***p < 0.001, ns, not significant.” appear below.

**Figure 6:**
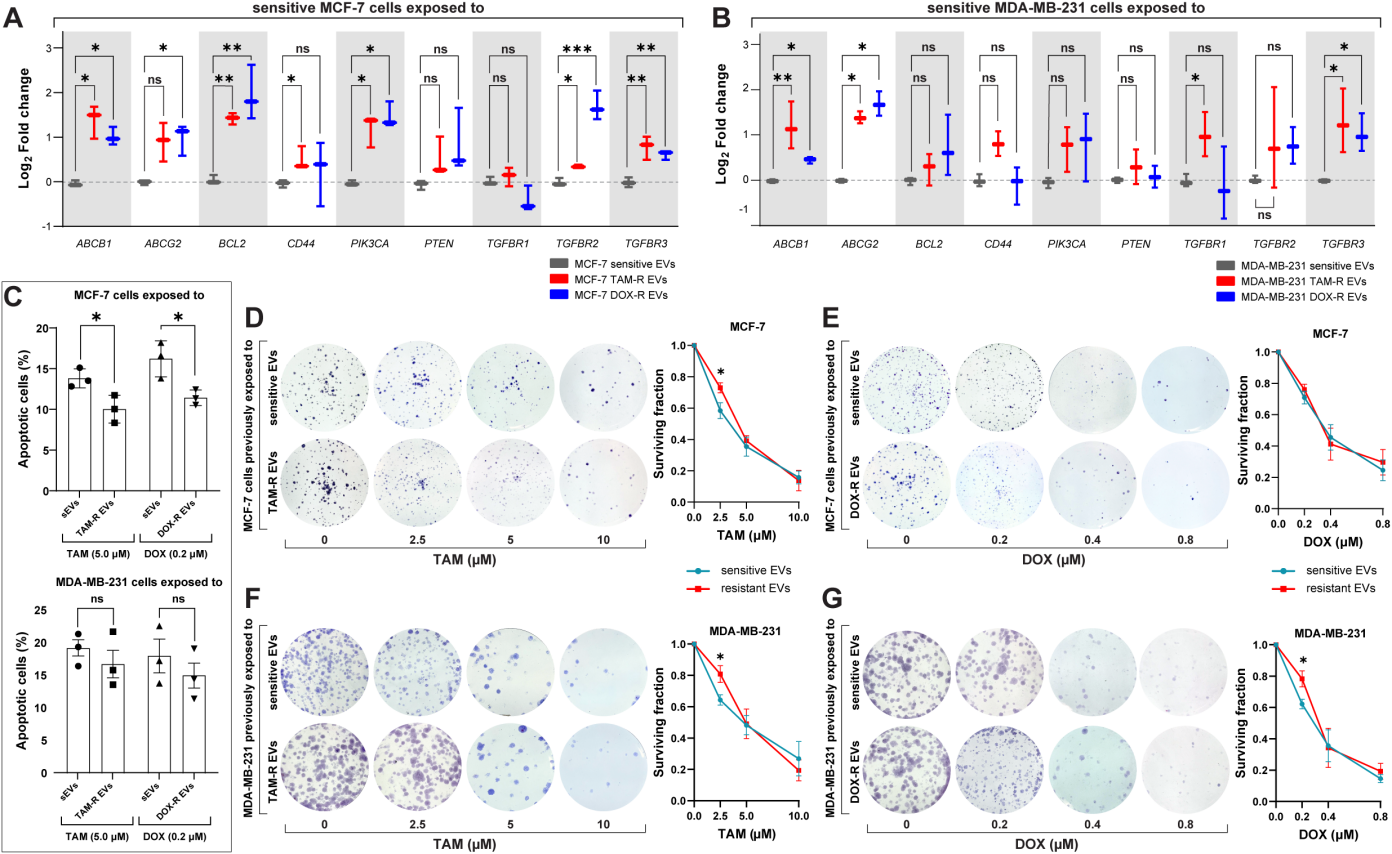
Resistant extracellular vesicles (EVs) induce the upregulation of genes associated with acquired drug resistance and increase the sensitive cells’ survivability. The quantification of several genes by quantitative polymerase chain reaction (qPCR) of **(A)** sensitive MCF-7 exposed to tamoxifen-resistant (TAM-R) and doxorubicin-resistant (DOX-R) EVs. **(B)** The gene expression level of sensitive MDA-MB-231 cells exposed to TAM-R and DOX-R. **(C)** The apoptotic rate by annexin V/propidium iodide labeling in sensitive MCF-7 and sensitive MDA-MB-231 previously exposed to resistant EVs and treated with TAM and DOX. **(D)** Representative images and the clonogenic surviving fraction of MCF-7 cells previously exposed to TAM-R and **(E)** DOX-R EVs treated with the respective drug. **(F)** Representative images and the clonogenic surviving fraction of MDA-MB-231 cells previously exposed to TAM-R and **(G)** DOX-R EVs treated with the respective drug. Values are displayed as the mean ± standard deviation from three independent experiments. The asterisks indicate a significant difference between TAM-R EVs or DOX-R EVs exposure compared to sensitive EV effects (unpaired t-test). *p < 0.05, **p < 0.01, ***p < 0.001, ns, not significant..

The authors apologize for this error and state that this does not change the scientific conclusions of the article in any way. The original article has been updated.

