# Supplementary material for: Extracellular vesicles from human breast cancer-resistant cells promote acquired drug resistance and pro-inflammatory macrophage response
Source: Front Immunol. 2024 Oct 15;15:1468229. doi: 10.3389/fimmu.2024.1468229 (PMC11518763; doi:10.3389/fimmu.2024.1468229)
Supplement: Supplementary file 1 [file Table1.docx]

Supplementary Material

# Supplementary Material 1

The alignment rate and the total of mapped reads of breast cancer RNA samples used for sequencing.

| Sample | nº of reads | Mapping (%) |
| --- | --- | --- |
| Sensitive MCF-7 (1) | 58.579.584 | 85,03 |
| Sensitive MCF-7 (2) | 54.780.420 | 84,86 |
| Sensitive MCF-7 (3) | 64.140.511 | 84,87 |
| DOX MCF-7 (1) | 54.022.025 | 72,30 |
| DOX MCF-7 (2) | 53.968.297 | 75,70 |
| DOX MCF-7 (3) | 66.952.682 | 72,13 |
| TAM MCF-7 (1) | 59.797.698 | 70,08 |
| TAM MCF-7 (2) | 59.027.129 | 81,90 |
| TAM MCF-7 (3) | 65.494.018 | 77,35 |
| Sensitive MDA-MB-231 (1) | 62.757.215 | 86,10 |
| Sensitive MDA-MB-231 (2) | 61.731.430 | 80,10 |
| Sensitive MDA-MB-231 (3) | 60.400.955 | 79,97 |
| DOX MDA-MB-231 (1) | 58.954.734 | 73,22 |
| DOX MDA-MB-231 (2) | 59.268.918 | 71,14 |
| DOX MDA-MB-231 (3) | 65.515.385 | 74,50 |
| TAM MDA-MB-231 (1) | 60.192.132 | 75,36 |
| TAM MDA-MB-231 (2) | 71.254.792 | 81,82 |
| TAM MDA-MB-231 (3) | 69.426.243 | 73,13 |

Supplemental Material 2:

List of human primers used for evaluation of gene expression. Tm°: Melting temperature.

| Gene | Sense | Sequence (5’-3’) | Tm° |
| --- | --- | --- | --- |
| *GAPDH* | Forward | TCGGAGTCAACGGATTTG | 62.4 |
|  | Reverse | CAACAATATCCATTTACCAGAG | 53.0 |
| *ABCB1* | Forward | AGTGAAAAGGTTGTCCAAG | 56.7 |
|  | Reverse | AGTCTGCATTCTGGATGG | 58.7 |
| *ABCG2* | Forward | AAAGCCACAGAGATCATAGAG | 57.6 |
|  | Reverse | GATCTTCTTCTTCTTCTCACC | 56.1 |
| *BCL2* | Forward | GATTGTGGCCTTCTTTGAG | 59.8 |
|  | Reverse | GTTCCACAAAGGCATCC | 59.0 |
| *CD44* | Forward | TTATCAGGAGACCAAGACAC | 56.6 |
|  | Reverse | ATCAGCCATTCTGGAATTTG | 61.5 |
| *PIK3CA* | Forward | GAGTAACAGACTAGCTAGAGAC | 50.8 |
|  | Reverse | AGAAAATCTTTCTCCTGCTC | 56.7 |
| *PTEN* | Forward | GGCTAAGTGAAGATGACAATC | 57.8 |
|  | Reverse | GTTACTCCCTTTTTGTCTCTG | 57.0 |
| *TGFBR1* | Forward | AGACAATGGCTTGGACTC | 55.5 |
|  | Reverse | GTACCAACAATCTCATGTG | 57.8 |
| *TGFBR2* | Forward | GGAGAAAGAATGACGAGAAC | 57.9 |
|  | Reverse | AGATGATGTTGTCATTGCAC | 58.5 |
| *TGFBR3* | Forward | ACCAAATCCAATTTCTCCAC | 60.2 |
|  | Reverse | GTGTGAGAATAGAGTACCAC | 53.3 |
